# Aortic “Disease-in-a-Dish”: Mechanistic Insights and Drug Development Using iPSC-Based Disease Modeling

**DOI:** 10.3389/fcell.2020.550504

**Published:** 2020-10-28

**Authors:** Hongorzul Davaapil, Deeti K. Shetty, Sanjay Sinha

**Affiliations:** Wellcome-MRC Cambridge Stem Cell Institute, Jeffrey Cheah Biomedical Centre, Cambridge, United Kingdom

**Keywords:** induced pluripotent stem cell, aortic aneurysm, Marfan, Loeys-Dietz, vascular smooth muscle, disease-in-a-dish

## Abstract

Thoracic aortic diseases, whether sporadic or due to a genetic disorder such as Marfan syndrome, lack effective medical therapies, with limited translation of treatments that are highly successful in mouse models into the clinic. Patient-derived induced pluripotent stem cells (iPSCs) offer the opportunity to establish new human models of aortic diseases. Here we review the power and potential of these systems to identify cellular and molecular mechanisms underlying disease and discuss recent advances, such as gene editing, and smooth muscle cell embryonic lineage. In particular, we discuss the practical aspects of vascular smooth muscle cell derivation and characterization, and provide our personal insights into the challenges and limitations of this approach. Future applications, such as genotype-phenotype association, drug screening, and precision medicine are discussed. We propose that iPSC-derived aortic disease models could guide future clinical trials via “clinical-trials-in-a-dish”, thus paving the way for new and improved therapies for patients.

## Introduction

Thoracic aortic disease usually proceeds silently until presenting suddenly with dissection or rupture (Pinard et al., 2019). Despite the frequently catastrophic and life-threatening consequences, there are no proven medical treatments for thoracic aortic disease beyond blood pressure control. Surgical replacement of the diseased section of aorta, either emergent or prophylactically, can be associated with significant morbidity and does not prevent disease progression or re-presentation in the non-replaced parts of the vessel. The lack of effective medical therapies has highlighted the critical need to define the mechanisms underlying aortic dilatation and dissection to inform the development of new treatments (Milewicz et al., 2005).

In contrast to abdominal aortic aneurysms, which have been shown to have links to inflammation and atherosclerosis, thoracic aortic aneurysms and disorders are frequently due to genetic factors (Humphrey et al., 2015; Pinard et al., 2019). A key question is to what extent the different genetic syndromes and disorders have common disease-causing pathways. The underlying mechanisms leading to aortic disease are still unclear despite the use of several mouse models; indeed, therapeutic discoveries made using the mouse models have not yet been shown to be effective in patients. Consequently, there is a pressing need for further studies and a wider range of model systems that may more fully predict a clinical response.

Through their seminal discovery of induced pluripotent stem cells (iPSCs), Takahashi and Yamanaka have bestowed the tools to now establish patient-derived complex models of human genetic diseases (Takahashi and Yamanaka, 2006). The power of this approach lies in the fact that these cells contain the patient’s DNA, so exhibit both the causal genetic defects as well as the permissive genetic background that allows florid disease presentation. Furthermore, these cells represent a versatile and almost unlimited resource for the study of early disease processes and for drug discovery. Such is their potential utility for understanding and treating diseases that they have been referred to as “disease-in-a-dish” models (Tiscornia et al., 2011).

In this review we will critically discuss recent studies where iPSCs have been used to model thoracic aortic aneurysm and dissection (TAAD) disorders. Since these related disorders have already been reviewed in detail by others (Goldfinger et al., 2014; Michel et al., 2018), we will only briefly cover the diseases themselves and highlight the controversies and major questions that have emerged in this field. We will then devote the majority of this review to providing insights into the practical aspects, applications, strengths, and limitations of using iPSCs to model these conditions. Finally, we will explore potential future directions for this approach including precision medicine and “clinical-trials-in-a-dish.”

## Aortopathies, Current Scientific, and Clinical Challenges

### Thoracic Aortic Aneurysm and Dissection

Thoracic aortic aneurysm and dissections commonly occur sporadically or in association with bicuspid aortic valves (BAV). Single gene disorders also cause thoracic aortopathies, notably in genes encoding extracellular matrix (ECM) components, transforming growth factor (TGF)-β signaling, or vascular smooth muscle cell (VSMC) contractile machinery (Brownstein et al., 2018). Marfan syndrome (MFS), caused by mutations in *FBN1*, is the commonest and best studied genetic disease resulting in TAAD. Other syndromic disorders include Loeys-Dietz syndrome (LDS) and vascular Ehlers-Danlos syndrome (vEDS) which are caused by mutations in the TGF-β signaling cascade (Lindsay et al., 2012) and in *COL3A1* (Pepin et al., 2000), respectively. Mechanistically, it is likely that TAADs share common disease mechanisms. Improving our understanding of Mendelian genetic disorders is also likely to lead to effective treatments for sporadic and bicuspid valve-associated aortopathies.

Many TAAD disorders show considerable overlap in pathology with elevated matrix metalloproteinases (MMPs), elastin fiber breaks, proteoglycan, and glycosaminoglycan deposition and medial aortic VSMC loss, suggesting common final pathways for aneurysm development despite varying genetic causes. An intimal tear then leads to an influx of blood and medial dissection; a condition with a cumulative 1% mortality per hour if the dissection involves the ascending aorta – a type A dissection (Anagnostopoulos et al., 1972). This dramatic surgical emergency is due to the propensity of a type A dissection to progress retrogradely and involve the coronaries, leading to myocardial infarction, or the pericardium, leading to tamponade. The risk of dissection is in part a function of aneurysm size, although the correlation varies widely depending on the precise disease as well as other familial factors and co-morbidities such as the presence of hypertension. Notably, some disorders such as LDS or vEDS, can present with arterial dissection or rupture at relatively normal vessel dimensions (Pepin et al., 2000; Williams et al., 2007), emphasizing the need for additional prognostic markers to supplement cross-sectional imaging.

In this review, we use MFS as the exemplar for genetically mediated TAADs. We will discuss the biological controversies and clinical issues raised by MFS to illustrate the challenges in the management of patients with TAAD and areas where novel approaches may be helpful. MFS is an autosomal dominant, multi-system disease affecting approximately 1 in 5000 people, caused by mutations in the gene encoding fibrillin-1, a key connective tissue ECM protein (Dietz et al., 1991). Fibrillin-1 glycoproteins assemble into microfibrils, which have both structural and functional roles. These microfibrils provide elasticity and provide a template for elastin fiber formation, but can also regulate the bioavailability of growth factors, such as TGF-β (Chaudhry et al., 2007), and provide attachment motifs for cell-matrix interactions (Kielty et al., 1992; Bax et al., 2003).

The cardiovascular complications are potentially fatal, and affect men more strongly than women (Murdoch et al., 1972; Pyeritz and KcKusick, 1979). Patients can develop mitral valve prolapse and aortic regurgitation, with the significant complication being aortic dilatation. These aortic aneurysms typically form in the aortic root and arch, and predispose to rupture or dissection (Milewicz et al., 2005). As with other TAADs, VSMCs from MFS patients typically have high expression and activity of MMPs, elastic fiber fragmentation and VSMC death, which all lead to weakening of the aortic wall (Segura et al., 1998; Ikonomidis et al., 2006; Grewal and Gittenberger-de Groot, 2018). In addition, there is increased deposition of collagen and proteoglycans, which contributes to increased vessel stiffness (Andreotti et al., 1985; Cattell et al., 1994). Indeed, patients with MFS tend to have stiffer aortas compared to the general population (Jeremy et al., 1994; De Wit et al., 2013; Hannuksela et al., 2018).

Mouse models of MFS have been very useful to understand a variety of disease aspects. Two models are commonly reported in the literature – the *FBN1*^C1039G/+^ (Judge et al., 2004) and *FBN1*^MgR/MgR^ (Pereira et al., 1999) models which represent moderate and severe disease, respectively. In addition to powerful tools to dissect the genetics, mouse models allow for deep phenotypic and histological characterization. The different stages of disease progression can also be investigated, making murine models essential for understanding disease mechanisms. The findings from these disease models, in addition to their drawbacks, will be discussed further below.

**FIGURE 1:**
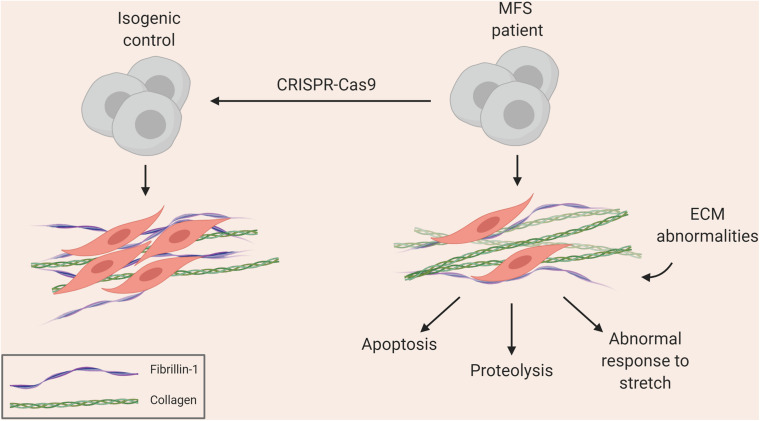
Summary of aortic disease phenotype recapitulated in MFS iPSC model (Granata et al., 2017).

Groups, including ours, have used iPSCs to investigate the pathology underlying MFS. Longaker and colleagues used a MFS embryonic stem cell (ESC) line as well as patient-derived iPSCs to show how antagonism of BMP signaling by TGF-β signaling impaired osteogenesis, leading to abnormal skeletogenesis (Quarto et al., 2012a, b). More recently, we used patient-derived iPSCs differentiated into VSMCs to recapitulate many aspects of vascular disease found in patients (Granata et al., 2017). This included increased MMP expression and cell death, fragmentation of ECM microfibrils, and reduced proliferation (Figure 1). Interestingly, when cells were exposed to cyclic mechanical stretch, the disease phenotype was further exaggerated, suggesting that there are abnormalities in mechanosensing/transduction, in line with current thinking about the mechanisms leading to disease progression. These disease features were rescued by using CRISPR-Cas9 mediated single nucleotide correction resulting in an isogenic normal control. iPSC-based models of other aortic diseases have also been developed, and are summarized in Tables 1, 2. These models have successfully recapitulated key aspects of aortic diseases, and have enabled identification of potential disease mechanisms for further investigation.

**TABLE 1:** Overview of current aortic disease models.

Disease model	Number of patient lines	Controls used (number of lines; clones)	Outcome
MFS Granata et al., 2017	2	Healthy iPSC (3) Isogenic control (1)	Characterization of model; identification of disease mechanism
LDS Gong et al., 2020	1; mutation introduced into wild-type line	Healthy iPSC (1); isogenic to mutant line	Characterization of model; preliminary 3D model
BVS Jiao et al., 2016	2	Healthy iPSC (2)	Characterization of model; identification of disease mechanism
SVAS Ge et al., 2012	1; 2 clones	Healthy iPSC (1; 2 clones)	Characterization of model; identification of disease mechanism
SVAS Kinnear et al., 2013	1; 4 clones	Healthy iPSC (1; 2 clones)	Characterization of model; identification of disease mechanism
SVAS Kinnear et al., 2020	5	Healthy iPSC (3)	Further characterization of model; preliminary 3D model; drug screen
SVAS Dash et al., 2016	1	Healthy iPSC (1)	Preliminary 3D model
HGP Zhang et al., 2014	2	Healthy iPSC (1)	Characterization of model; identification of disease mechanism
HGP Liu et al., 2011	1	Healthy iPSC (1)	Characterization of model; identification of disease mechanism
HGP Zhang et al., 2011	2	Clinically normal parent iPSC (2)	Characterization of model
HGP Atchison et al., 2020	2	Healthy iPSC (2)	Characterization of 3D model

**TABLE 2:** Summary of the differentiation protocols and parameters in aortic disease models.

Protocol ref.	Use in disease modeling	Method	Length of VSMC induction	Media for VSMC induction	Markers of VSMCs detected	% Marker Expression	Contractility (time of assessment)	Lineage-specificity
Cheung et al., 2012, 2014; Serrano et al., 2019	MFS Granata et al., 2017	Monolayer through embryonic intermediates	12 days and 30 days maturation	TGF-β (2 ng/ml) PDGF-BB (10 ng/ml); 10% FBS	*ACTA2, CNN1, TAGLN, SMTN MYH11*	>80% double-positive for *MYH11* and *CNN1* by FC	Carbachol (3 min)	NC, LM, and PM
Modification of Patsch et al., 2015 for CPC-VSMCs Modification of Mica et al., 2013; Xiong et al., 2017 for NC-VSMCs	LDS Gong et al., 2020	Monolayer through embryonic intermediates	For CPC-VSMCs: 6 days For NC-VSMCs: 8 days	For CPC-VSMCs: TGF-β1 (2 ng/ml) PDGF-BB (10 ng/ml) For NC-VSMCs: 20% KSR TGF-β (2 ng/ml)	*ACTA2, CNN1, TAGLN, SMTN MYH11*	Expression detected by qPCR and western blotting	Carbachol (30 min)	Cardiovascular progenitor cell (LM) and NC
Jiao et al., 2016	BVS Jiao et al., 2016	Monolayer through embryonic intermediates	9 days	15% KSR TGF-β (2 ng/ml)	*ACTA2, CNN1, TAGLN, MYH11*	>70% positive for *MYH11* by FC	Carbachol (30 min)	NC and PM
Xie et al., 2007	SVAS Ge et al., 2012; Kinnear et al., 2013, 2020	EB	5–12 days	SMGM (Lonza); 5% FBS	*ACTA2, CNN1, TAGLN, MYOCD, MYLK, SMTN, MYH11*	55% positive for *MYH11*; 97% positive for *ACTA2* by FC	Carbachol (30 min)	NR
Modification of Xie et al., 2007	SVAS Dash et al., 2016	EB	17 days	SMGM-2 (Lonza); 0.5% FBS TGF-β (1 ng/ml)	*ACTA2, CNN1, TAGLN, ELN, MYH11*	87% positive for *MYH11*; 75% positive for *ELN* by FC	Carbachol and KCl (15 min)	LM; inferred from cytokine response
Modification of Xie et al., 2007	HGP Zhang et al., 2014	EB	42 days	SMGM (Lonza); 5% FBS	*ACTA2, CNN1, TAGLN*	>80% double positive for *ACTA2* and *CNN1* by FC	Angiotensin II (30 min)	NR
Liu et al., 2011	HGP Liu et al., 2011	Monolayer through CD34^+^ progenitor	NR	SMGM-2 (Lonza)	*ACTA2, CNN1*	NR	NR	NR
Modification of Jeon et al., 2006	HGP Zhang et al., 2011	EB-derived mesenchymal stem cell (MSC)	3 weeks	SPC (5 mM) TGF-β (2 ng/ml)	*ACTA2, CNN1, TAGLN, SMTN, MYH11*	50–60% positive for *MYH11* by IF	Carbachol (60 min)	Mesoderm
Modification of Patsch et al., 2015	HGP Atchison et al., 2020	Monolayer through embryonic intermediate	6 days	Activin A (2 ng/ml) PDGF-BB (10 ng/ml) Heparin (2 μg/ml)	*ACTA2, CNN1, MYH11*	>99% positive for *MYH11* by IF >90% positive for *ACTA2* and *CNN1* by FC	U46619 (10 min)	Mesoderm

*MSC, Mesenchymal stem cell; KSR, Knock-out Serum Replacement; SMGM, Smooth Muscle Growth Medium; SPC, Sphingosylphosphorylcholine; FC, Flow Cytometry; IF, Immunofluorescence; and NR, not reported.*

### TGF-β Controversy – Cause or Consequence?

The TGF-β signaling pathway is crucial for normal VSMC function and it is a potent cytokine regulating proliferation, differentiation, extracellular matrix remodeling, and apoptosis (Guo, 2012). Activation of TGF-β receptors leads to canonical signaling through Smads but also non-canonical signaling through MAPKs (Zhang, 2017). Analysis of the lung of a severe mouse model of MFS, *FBN1*^mgΔ^, found increased activation of TGF-β (Neptune et al., 2003). Since treatment with a TGF-β neutralizing antibody rescued the lung phenotype, the Dietz lab hypothesized that the loss of microfibrils decreased the sequestration of TGF-β and in turn led to an increase in local TGF-β signaling. This line of thinking was supported by findings in the moderate *FBN1*^C1039G/+^ murine model (Judge et al., 2004), where increased canonical TGF-β signaling was detected in the dilated aorta. Treatment with a TGF-β neutralizing antibody once again rescued the disease phenotype (Habashi et al., 2006), as did blockade of the angiotensin II receptor type 1 (AT1R) with losartan, which reduced TGF-β expression and non-canonical signaling (Lavoie et al., 2005; Rodríguez-Vita et al., 2005; Holm et al., 2011).

Given the dramatic results with losartan in mouse models of MFS, a series of clinical trials in patients commenced. An initial retrospective analysis of a pediatric cohort of MFS patients suggested promising results in slowing aortic dilatation (Brooke et al., 2008). Several randomized trials have now been carried out comparing losartan either to β-blocker or to placebo (Groenink et al., 2013; Lacro et al., 2014; Milleron et al., 2015; Teixido-Tura et al., 2018). Surprisingly, despite some early promise in small trials, the largest single randomized study has shown that losartan had no statistically significant improvement in children and young adult patients when compared to β-blockers (Lacro et al., 2014). Related to the findings in the initial retrospective analysis, this larger study found that the younger subjects were more responsive to treatment with losartan compared to the older cohort, suggesting that there may be an early therapeutic window for targeting angiotensin II signaling.

Subsequent evidence from mouse studies has indicated that the nature of TGF-β signaling in TAAD progression is complex, and may also confer a protective effect. Post-natal VSMC-specific deletion of TGF-β receptor II (TβRII; Hu et al., 2015) or treatment with a TGF-β neutralizing antibody (Wang et al., 2010) led to severe aortopathy. Indeed, crossing *FBN1*^C1039G/+^ mice with a conditional knock-out for *Tgfbr2* exacerbated the aortic phenotype, indicating that TGF-β may have a protective effect (Li et al., 2014; Wei et al., 2017). *FBN1*^MgR/MgR^ is a severe model for MFS (Pereira et al., 1999) in which treatment with losartan slightly improved lifespan, but did not have the same impact as in the moderate *FBN1*^C1039G/+^ model (Xiong et al., 2012; Cook et al., 2015). In addition, treatment with a TGF-β neutralizing antibody was detrimental at P16, but beneficial at P45, indicative of a temporally dependent role for TGF-β in aneurysm formation (Cook et al., 2015). Other studies did not find any benefit of TGF-β or angiotensin II signaling inhibition in VSMCs (Angelov et al., 2017; Galatioto et al., 2018). Together, these lines of evidence indicate that the pathophysiology of MFS is more complex than just dysfunction of TGF-β signaling in VSMCs. The upregulation of TGF-β signaling in MFS may in part be a compensatory mechanism, rationalizing the increase observed in patients with severe aneurysm (Franken et al., 2013).

The losartan and TGF-β controversy indicates that further mechanistic validation is required when transitioning between mouse studies and patient treatment, particularly in the context of the human genome. While losartan was highly-effective and promising in a mouse model, its effectiveness was not matched in patients. This was potentially due to fundamental differences in the anatomy between murine and human aortas, but also due to the disparity between the dose required to elicit a response, and the dose deemed safe for human patients. Recently, another AT1R antagonist, irbesartan, was found to be effective in reducing aortic dilatation in children and young adults (Mullen et al., 2020). Although losartan and irbesartan both inhibit AT1R, irbesartan has greater bioavailability and a longer half-life, implying that the difference in outcome may be in part due to insufficient duration of action of losartan. In addition, while the mice used for the losartan studies were genetically homogeneous and treated at the same age, the human patients introduced variability via their disease-causing mutations, genetic backgrounds and ages at treatment. Although animal models allow us to study various stages of disease and are still needed to assess potential therapeutic targets, this case has highlighted the need for an additional platform to assess the viability of mechanisms and treatments in a variety of patient lines before applying them in the clinic.

### Abnormalities in Mechanosensing

If excess TGF-β signaling is not causal in MFS, then what is? The contractile machinery of VSMCs is composed of thin and thick filaments that contain α-smooth muscle actin (α-SMA; *ACTA2*) and smooth muscle myosin heavy chain (SM-MHC; *MYH11*), respectively. In healthy conditions, once stress has been sensed via integrins (Martinez-Lemus et al., 2003), VSMCs can secrete various factors such as MMPs, TGF-β and angiotensin II to adapt the ECM and modulate VSMC phenotype to maintain blood pressure homeostasis (O’Callaghan and Williams, 2000). TAAD-causing mutations in *ACTA2* and *MYH11* disrupt their function (Zhu et al., 2006; Guo et al., 2007), suggesting that reduced VSMC contractility may be an underlying disease mechanism. In an iPSC model of LDS, where a mutation in *SMAD3* was created, the resulting VSMCs had decreased expression of contractile markers (Gong et al., 2020). Similarly, ECM mutations may disrupt the VSMC linkage to the matrix and ability to accurately sense wall stress. This is supported by electron microscopy images from MFS mice showing abnormally smooth elastic fibers due to reduced VSMC attachment (Bunton et al., 2001). It has therefore been proposed that abnormalities in mechanosensing, erroneous ECM remodeling and cellular response lead to aneurysm formation (Humphrey et al., 2015; Pinard et al., 2019). Another way in which mechanical forces may act could be by reduced vascular tone resulting in increased interstitial fluid leading to the formation of intramural edema and dissection (Mallat et al., 2016). This is supported by a study in rat abdominal aortic rings, where noradrenaline-stimulated VSMC contraction decreased hydraulic conductance (Chooi et al., 2017).

The mechanosensing hypothesis is supported by evidence from mouse models. Endothelial nitric oxide (NO)-mediated vasodilation exacerbated aortic aneurysm (Oller et al., 2017). In addition, treatment with calcium channel blockers as an alternative to current anti-hypertensive drugs also accelerated aneurysm formation in a model of MFS (Doyle et al., 2015). Post-natal *Tgfbr2* knock-out in mice led to decreased contractile gene expression and compaction in a collagen gel assay (Li et al., 2014). Further assessment of these mice found compromised aortic mechanical properties compared to controls, and treatment of these animals with rapamycin restored some of these mechanical properties and prevented pressure-induced delamination *in vitro* (Ferruzzi et al., 2016). Rapamycin has been shown to improve VSMC contractility (Martin et al., 2004), and has been used to rescue VSMC de-differentiation phenotypes, including *in vitro* disease models of supravalvular aortic stenosis (SVAS) and BAV/TAA (Kinnear et al., 2013; Jiao et al., 2016).

The relationship between inappropriate mechanosensing and TAAD formation is not yet fully understood. In addition to using animal models, iPSC-derived VSMCs could be used to investigate this hypothesis, as they can be genetically modified and stretched using various cell-stretching apparatus. We observed worsening of the disease phenotype upon cyclic stretch in our *in vitro* model of MFS (Granata et al., 2017), indicating that current protocols result in VSMCs sufficiently mature to be capable of mechanotransduction. Substrate stiffness is also something which can be explored – as mentioned above, the aortas of patients with TAADs tend to be stiffer. Combining iPSC-based models with hydrogels of varying stiffnesses could provide insights into the role of vessel wall stiffness in aortic disease.

### Understanding the Early Stages of Aortic Disease

Samples of diseased aortas can only be obtained from late-stage disease at the time of surgery, thus providing markers and mechanistic insight corresponding to severe TAADs only. From a therapeutic stand-point, investigating late-stage tissue provides limited information for developing novel therapies to prevent progression or identifying biomarkers for various stages of disease. Another challenge of using tissue from patients is the lack of appropriate controls. It is highly unlikely that researchers can obtain clinical samples of a healthy individual’s aorta, but surgeons repairing a diseased aorta may collect biopsies from non-diseased sections, or at least from regions displaying no visible defects. However, such samples likely do not truly represent a healthy aorta, especially in the case of genetic disorders. Also, a region adjacent to the aneurysm could still exhibit defects in the ECM, signaling and response to mechanical stimuli. In addition, cytokines and growth factors in the circulation as well as local environmental cues may also contribute to the disease phenotype.

Early events in disease progression need to be better understood and characterized. As will be discussed further, there is significant variation in the disease presentation of MFS, even among individuals with the same causative mutation in *FBN1*. It is therefore difficult to predict from initial diagnosis whether disease progression will be mild or severe and this is a particular problem for sporadic cases with no family history. In addition, in disorders such as vEDS, patients do not tolerate surgery, with high post-operative mortality (Bergqvist et al., 2013). Consequently, treating patients at an early stage to prevent presentation or slow aneurysm growth would be ideal, and therefore understanding the early events in disease progression is critical.

These limitations may be circumvented by the use of iPSC-derived VSMCs. A virtually unlimited supply of cells can be generated from patient-derived iPSC lines along with genetically corrected isogenic controls. In our experience, both early and late events can be captured to some extent *in vitro*. For example, accumulation of disease phenotype with age is observed in the iPSC model of MFS. After differentiating MFS iPSCs to neural crest (NC)-derived VSMCs, we allow the cells to mature in serum for 30 days – during this time, the cells accrue a more severe phenotype in the dish, including increased proteolytic activity and apoptosis (Granata et al., 2017). We observed that NC-VSMCs at an earlier stage did not show the same intensity of disease characteristics, suggesting that, to an extent, we can mimic disease progression *in vitro*. We therefore suggest that iPSC-based models of VSMCs enable us to generate appropriate control cells and uncover events at various stages of disease progression.

### Conclusion

Thoracic aortic aneurysm and dissections are a group of disorders with life-threatening circumstances, and although surgical intervention has increased the mean life expectancy from 45 to 70 years in MFS (Milewicz et al., 2005), new medical treatments need to be urgently identified. Confounding results between mouse and clinical studies have emphasized the need for an additional assessment platform. iPSC-based modeling of aortic disease can be employed, where mechanistic and patient-specific information is used to direct future clinical trials and precision medicine. In the next section, we will discuss practical considerations for constructing a “disease-in-a-dish.”

## Practicalities of Aortic Disease Modeling

### What do We Look for?

*In vitro* differentiation protocols are generally founded on developmental principles (Keller, 2005; Ayoubi et al., 2017). For VSMC development, a huge body of work exists and as a detailed discussion is beyond the scope of this review, we refer the reader to excellent reviews written by others (Owens et al., 2004; Owens, 2007). Briefly, after endothelial cells (ECs) form a lumen mural cells are recruited and invested to stabilize the nascent vessel through various signaling axes, such as TGF-β, PDGF-BB, Notch and angiopoietin/Tie2 (Drake, 2003; Liu et al., 2009; Stenzel et al., 2009; Patel-Hett and D’Amore, 2011). This leads to the establishment of transcriptional modules, including SRF, GATA factors, and myocardin (Croissant et al., 1996; Manabe and Owens, 2001a; Chen et al., 2002; Nishida et al., 2002; Du et al., 2003). In addition, post-transcriptional processes, such as miR-143/145, have also been shown to contribute to this VSMC identity (Boucher et al., 2011). Finally, changes in the epigenome have been shown to allow binding of key transcription factors to their promoters, and lead to stabilization of this VSMC-specific gene expression, while still allowing for phenotypic plasticity depending on the integration of various inputs by the cells (Manabe and Owens, 2001b). Together, these processes lead to the stable expression of VSMC-specific gene expression. These markers of VSMCs can be used in stem cell-derived products to assess their identity and serve as a point for quality control.

An iPSC model is only as good as the differentiation protocol used. A variety of VSMC differentiation protocols exist and we have summarized those protocols that have been used in aortic disease modeling in Table 2; general VSMC differentiation protocols have been reviewed thoroughly by others (Ayoubi et al., 2017). When choosing a protocol to model aortic disease, there are a few parameters to consider. First, the length and nature of the protocol – older methods describe embryoid body (EB) differentiations, where aggregated stem cells spontaneously differentiate into the three germ layers, recapitulating events during development (Itskovitz-Eldor et al., 2000). From this point, VSMC fate can be induced. Differentiation through EBs requires precise control of cell aggregates, in respect of both size and homogeneity, as these can influence differentiation and yield (Messana et al., 2008), potentially due to cytokines and small molecules exerting their effects mainly on the surface layers (Sachlos and Auguste, 2008). Cell sorting by FACS could circumvent this issue, however, there are considerations for time and cell viability following sorting. Although methods have been developed to reduce variation in EB size and density, including the use of microwells, and micropatterned scaffolds (Bauwens et al., 2008; Mohr et al., 2010), the field has largely moved away from EBs to monolayer methods (Cheung et al., 2012; Mummery et al., 2012; Patsch et al., 2015; Palakkan et al., 2017). Generally, pluripotent stem cells grown as monolayer colonies are first directed toward a specific embryonic pathway, and then differentiated into VSMCs. This allows for more uniform delivery of factors guiding differentiation, as there are fewer considerations for factor diffusion and availability (Suchorska et al., 2017). Monolayer methods are also more amenable to large-scale production, due to their relative homogeneity compared to EBs, and do not necessarily require any cell sorting.

Another important consideration would be the presence of appropriate VSMC markers, indicative of maturation and contractility. With the possible exception of SM-MHC and smoothelin (*SMTN*), most VSMC markers can be expressed in other cell types under certain conditions (Alexander and Owens, 2012). Therefore, if the aim is to obtain relatively mature and contractile VSMCs, staining or flow cytometry of SM-MHC and/or smoothelin would be more appropriate ways of monitoring differentiation quality, rather than a less selective marker such as α-SMA. If opting for SM-MHC antibody staining, however, we caution readers to carefully assess the data – cross-reactivity of smooth muscle and non-muscle myosin heavy chains by polyclonal antibodies can confound interpretation of results and can lead to over-estimation of SM-MHC content (Rovner et al., 1986). It should be noted that *in vitro* differentiated cells can easily lose SM-MHC and smoothelin expression when exposed to serum (Alexander and Owens, 2012), so quality control to identify these VSMC markers should be performed prior to culture in serum. Furthermore, some patient-derived lines of familial TAADs may have mutations in VSMC contractile genes such as *MYH11* or *ACTA2*, so appropriate control lines, such as CRISPR-corrected isogenic lines, should be used in parallel in order to assess the quality of differentiations. In addition to marker expression, functional assays should also be performed. Identifying a protocol where the cells show VSMC-like responses, with rapid contraction, to vasoactive agonists such as carbachol would also be important to ensure that the correct cell type, or good differentiation, has been achieved. VSMC contraction should be noticeable on the scale of a few minutes, rather than hours (Table 2).

We appreciate that certain mutations will alter the expression of markers and function of resulting VSMCs. Care should be taken when establishing new disease models or lines to distinguish poor quality differentiations from genuine *in vitro* disease phenotypes. This may be particularly relevant in diseases or patient lines with mutations in genes affecting VSMC function, including the aforementioned *MYH11* or *ACTA2* mutations. In these cases, in addition to using gold standard isogenic controls, we strongly recommend careful and stringent quality control of the VSMC progenitor. This will reduce variability in the resulting VSMCs, and result in more consistent assessment of the disease phenotype.

### Maturity and Phenotype

Generating and analyzing contractile VSMCs is of crucial importance in recapitulating disease phenotypes. The importance of iPSC-derived maturity can be inferred firstly from the fact that TAADs are generally post-natal diseases rather than developmental. Moreover, with diseases related to VSMC de-differentiation, such as SVAS, restoration of full VSMC function and maturity *in vitro* would be an essential parameter of success for any new therapeutic. The inability of a differentiation protocol to yield mature VSMCs in control lines is likely to fatally compromise drug screening or testing with that protocol. In our experience, in addition to the specific protocol used, the contractile ability can be affected by user-dependent factors such as the seeding density during or after differentiation; these are important considerations as they contribute significantly to variation between differentiations, as will be highlighted below.

Vascular smooth muscle cell differentiation protocols can be further refined to improve the yield of contractile cells. In addition to reduction or replacement of serum in the maturation steps, small molecules can be introduced to improve yield of contractile VSMCs. Recently, a novel screening method was reported, where an *MYH11* reporter ESC line was used to screen over 4,000 compounds that may improve SM-MHC expression (Zhang et al., 2019). This screen identified RepSox, a modulator of Notch signaling, as improving VSMC contractility in differentiations using PDGF-BB and TGF-β. In addition to improvement in initial levels of SM-MHC, cells treated with RepSox also maintained high levels of SM-MHC for at least 8 weeks after derivation, suggesting that this may be a new and interesting direction for VSMC differentiation protocols.

### Lineages

**FIGURE 2:**
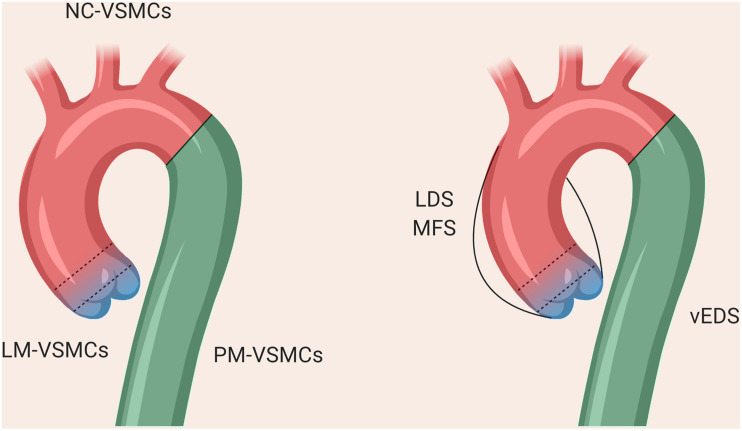
The different regions of the thoracic aorta and their disease susceptibilities. The descending aorta comprises VSMCs from paraxial mesoderm, the aortic arch from neural crest, and the aortic root from lateral plate mesoderm. The boundary between the arch and descending aorta is clearly defined, whereas there is overlap between the VSMCs from NC and LM at the aortic root, as denoted by the dotted lines.

The VSMCs comprising the aorta are derived from distinct embryonic lineages: the descending aorta is derived from paraxial mesoderm (PM), the ascending aorta and aortic arch from NC and the aortic root from lateral plate mesoderm (LM; Jiang et al., 2000; Wasteson et al., 2008; Harmon and Nakano, 2013; Figure 2). These different aortic regions seem to have distinct susceptibility to aortic diseases, including genetically-triggered aortopathies, suggesting that in addition to haemodynamics and wall structure, the embryonic lineage of the VSMC may be an important determinant for disease development and progression, [reviewed by (Majesky, 2007)]. In addition, the nature of the border between VSMCs of different lineages could be an important consideration; while there is a distinct boundary at the aortic isthmus between the PM- and NC-derived VSMCs (Nakamura et al., 2006), the transition between LM- and NC-VSMCs in the aortic root is not as well defined. Lineage-tracing experiments in mice have shown that there is a significant area of overlap between these lineages at the base of the aorta (Harmon and Nakano, 2013; Sawada et al., 2017). Indeed, it has been suggested that the differential response to cytokines and/or ECM composition between these overlapping or adjacent VSMC populations underpins the origins of aortic aneurysm and dissection (Topouzis and Majesky, 1996; Cheung et al., 2012), an hypothesis supported by recent work in mice (Angelov et al., 2017; MacFarlane et al., 2019). *Tgfbr2* deletion in VSMCs led to the development of thoracic aortic aneurysms, whereas treatment with a TGF-β neutralizing antibody resulted in abdominal aortic aneurysms (Angelov et al., 2017). Lineage tracking and sorting in a Loeys-Dietz mouse model showed a differential response of LM- and NC-derived VSMCs to TGF-β (MacFarlane et al., 2019).

As a result, protocols describing the derivation of VSMCs corresponding to the different regions of the aorta may be important to consider in order to accurately reflect the disease (Cheung et al., 2012; Patsch et al., 2015; Jiao et al., 2016; Gong et al., 2020). Our *in vitro* model of MFS showed differences in fibrillin-1 deposition and disease severity in VSMCs depending on embryonic origin, highlighting the importance of studying specific cohorts of VSMCs when modeling a “disease-in-a-dish” (Granata et al., 2017). A model of BAV has also demonstrated that NC-VSMCs, but not PM-VSMCs, from patients with BAV and TAA have defects in differentiation and contractile function (Jiao et al., 2016). Currently published iPSC models for SVAS, however, did not use lineage-specific protocols in their investigation (Ge et al., 2012; Kinnear et al., 2013, 2020). In a 3D model of SVAS, a lineage-specific protocol also was not used, although the investigators inferred lineage based on responsiveness to cytokines (Dash et al., 2016). Finally, in a recent iPSC-based model of LDS, NC-, and LM-VSMCs exhibited distinct defects relating to contractile marker expression and response to TGF-β depending on lineage (Gong et al., 2020), mirroring the *in vivo* findings (MacFarlane et al., 2019). These studies collectively echo the importance of using lineage-specific protocols wherever possible when modeling aortic disease.

### Contraction and Response to Stretch

VSMC contractility in response to agonists is an important indicator of maturity and this can be assayed in 2D and 3D systems. Contraction of VSMCs can be examined upon exposure to either ionophore compounds such as potassium chloride, ionomycin, or carbachol or peptide hormones such as angiotensin II. Angiotensin II activates AT1R, stimulating a cascade of G-coupled protein signaling or tyrosine phosphorylation triggering MAPK signaling followed by intracellular calcium release, which leads to contraction (Griendling et al., 1997; Touyz and Schiffrin, 1997). The extent of contraction can be investigated by comparing cell surface area before and after agonist stimulation, or more sophisticated methods involving live-imaging and/or force measurements can be employed (Gaio et al., 2016; Halaidych et al., 2019; van Meer et al., 2019). Importantly, routine examination of contractile response should be assayed in iPSC-derived VSMCs to ensure the consistency of differentiations.

In addition to being a functional benchmark, contraction has the ability to drive maturation. VSMCs contract to counterbalance hemodynamic forces as well as circumferential strain in blood vessels and, in response to these, maintain blood flow and pressure (Zulliger et al., 2004; Alexander and Owens, 2012; Ahmadzadeh et al., 2019). Pulsatile stretch is interpreted by cells through intracellular signaling pathways leading to changes in proliferation, contraction, apoptosis, migration, and ECM remodeling (Haga et al., 2007). VSMC contraction does not only define the maturity of these cells, but the application of uniaxial mechanical forces using stretching platforms can itself induce functional differentiation of the nascent iPSC-derived VSMCs. Cyclic stretch is applied to VSMCs seeded on ECM-coated elastomer-bottomed culture plates and, over 6 to 48 h, the VSMCs align themselves based on the strain cues (Mantella et al., 2015). Stretched VSMCs have synchronized contraction and increased myocardin expression, indicative of enhanced contractility (Zhu et al., 2011; Raphel et al., 2012; Chiu et al., 2013; Qiu et al., 2013). It should be noted that uniaxial stretch promotes VSMC differentiation whereas equiaxial stretch has the opposite effect (Park et al., 2004) therefore the choice of method needs careful consideration.

Another mode of enhancing contractility is by the use of pulsatile flow, which has proved to be effective in improving both VSMC alignment and contractility in 2D as well as 3D culture systems (Shi and Tarbell, 2011). Cyclic stretch aided alignment of VSMCs and deposition of elastin as well as other ECM components such as collagen, which in turn enhanced tensile strength and elasticity of scaffolds, vascular rings and tissue engineered blood vessels (TEBVs) made of VSMCs and ECs (Solan et al., 2009; Cooper et al., 2014). Here, the stretched constructs demonstrated higher burst strength and elasticity compared to non-stretched counterparts, making them both more amenable for *in vivo* transplant and a more accurate disease model *in vitro*.

These simple 3D models are amenable to contractility assays and can supplement standard 2D *in vitro* systems. In addition, they offer the possibility to test VSMC interactions with other cell types such as ECs and fibroblasts (Jung et al., 2015; Ding-Yang et al., 2019). VSMCs embedded in collagen or Matrigel have been shown to reorganize and remodel their environment to more closely mimic *in vivo* ECM architecture (Song et al., 2001; van den Akker et al., 2012). Contraction can also be assayed in these 3D systems, which more closely resemble native blood vessels than 2D cultures. The collagen gel contraction assay is a typical one to assess functionality of VSMCs by measuring the reduction in gel area and has been applied to both primary and stem cell-derived VSMCs (Oishi et al., 2000; Sinha et al., 2006; van den Akker et al., 2012; Lee et al., 2019). Newer models employ the use of bioreactors for scale-up, and 3D hydrogel disks are prepared by mixing multiple cell types like VSMCs and ECs with collagen, and contraction assayed over 30 min to 1 h (Lin et al., 2019). Vascular rings, a 3D structure comprising VSMCs, can be created relatively quickly and changes in circumference or force generation can be assayed in response to contractile agonists (Bi et al., 2005; Dash et al., 2016). Dash and colleagues have successfully created rings using iPSC-derived VSMCs to create a preliminary 3D model of SVAS. Here, the vascular rings created from patient VSMCs exhibited reduced contractility, which was a similar finding to previously published 2D models of SVAS with the strength of analyzing collective force generation and contraction versus single cells in a monolayer (Ge et al., 2012; Kinnear et al., 2013).

An important consideration for aortic disease modeling is that the full of extent of VSMC dysfunction may not be evident in unstretched or unstimulated circumstances. For example, there may be defects in contraction or contractile responses which are critical for the disease phenotype which are not otherwise apparent. Subjecting cells to mechanical forces would emulate the *in vivo* strain, as well as triggering associated signaling pathways, such as the generation of physiological reactive oxygen species (Clempus and Griendling, 2006). This was recently highlighted by a study in vEDS mouse models, where the differences in collagen organization were only apparent after stretching (Dubacher et al., 2020). When developing new therapies for aortic disease, it is essential to ensure that the disease effect on VSMC contraction and mechanotransduction are sufficiently evaluated.

### Gene Editing to Create Isogenic Controls

With the advances in tools for gene editing, the use of isogenic controls is now the gold-standard in iPSC modeling. Many stem cell banks have catalogs of extensively-characterized healthy iPSC lines and these can be used as controls compared to patient lines. However, diseases such as MFS have high inter- and intra-familial variability – the same mutation in *FBN1* can result in varied disease presentations (Dietz et al., 1992). Consequently, gene editing, to provide a “corrected” wild-type version of the disease line, offers the significant advantage of an isogenic control line that has the same genetic background as the disease model but differs only by the few nucleotides that constitute the mutation. Although this approach is widely-used in many fields (Bassett, 2017), current aortic disease models mainly rely on healthy iPSC lines as controls (Table 1).

Of course, the gene editing tools used to correct a mutation can easily be used to create a mutation in an otherwise healthy control iPSC line. Several groups have used this approach to generate disease models without needing patient involvement (Paquet et al., 2016; Tidball et al., 2017; Frederiksen et al., 2019), including a recent model for LDS (Gong et al., 2020). Despite the obvious practical advantages of this strategy, we should sound a note of caution. If there is any variable expressivity of the mutation, then a permissive genetic background may be required for full disease manifestation *in vitro*, which unlike lines from patients with disease, is uncertain in healthy control iPSC lines. We predict that creating a patient mutation in a healthy line will not necessarily yield the same extent of cellular defects as the patient line. This will be particularly important for multi-variant disorders, but also when modeling disease from patients with milder clinical manifestations. In the case of the monogenic aortic diseases discussed here, genetic background likely plays an important role in influencing disease severity and presentation, as will be discussed later. In practical terms, in order to construct an accurate “disease-in-a-dish,” we recommend the use of patient lines and genetically-engineered isogenic controls as the gold standard. Alternatively, wherever possible, iPSCs from unaffected family members could also be used as controls, which partly mitigates the differences in genetic backgrounds. In a model of Hutchison-Gilford progeria (HGP), researchers obtained unaffected parental fibroblasts in addition to patient lines from the Coriell Institute cell bank (Zhang et al., 2011); unfortunately, in this case, parents and patients were from different families.

### Conclusion

Many differentiation protocols exist for producing VSMCs and the choice of protocol can have important effects on the quality of disease modeling. This may be particularly pertinent in modeling aortic disease, as three lineages of VSMC are present in the aorta and may be involved with disease susceptibility, but also because aortopathies may result from improper VSMC function, such as abnormal proliferation and contractility. Once differentiated, iPSC-derived VSMCs provide a flexible system to address aspects of a disease – simple cell-based assays such as the assessment of proteolytic activity, proliferation, contractility, and response to mechanical stimuli can provide mechanistic insight. Lastly, gene editing tools allow researchers to create virtually any genetic modification in their patient-derived or healthy lines, creating opportunities to untangle issues such as the genotype-phenotype correlation in TAADs. Despite these advantages, there are a number of issues to be aware of which we will discuss next.

## Limitations of Current Approaches to Aortic Disease Modeling

### Production of Immature Cells

Cell maturity is a major consideration with iPSC-based modeling of aortic disease. Current iPSC differentiation protocols almost invariably result in cells which are closer to fetal VSMCs than to adult cells, as has been demonstrated in other fields (Mummery et al., 2012; Lundy et al., 2013; Hrvatin et al., 2014; Baxter et al., 2015). While this immaturity has been best characterized in cardiomyocyte and hepatic differentiation, a similar problem is likely to exist in VSMC differentiation; although the exact developmental stage, perhaps due to intrinsic VSMC plasticity (Alexander and Owens, 2012), is poorly characterized in most VSMC studies. Nevertheless, low levels of SM-MHC and smoothelin expression confirm that these iPSC-VSMCs are most likely to represent a fetal-like state. While this may be advantageous for developmental studies and disorders, caution is warranted for adult disease modeling and the potential drawbacks have been discussed earlier. It is possible to improve the maturity of the *in vitro* derived VSMCs using a range of strategies including EC co-culture (Collado et al., 2017), application of mechanical force (Park et al., 2004; Ghazanfari et al., 2009), small molecules or other growth factors such as TGF-β and retinoic acid (Martin et al., 2004; Yu et al., 2011; Wanjare et al., 2013; Zhang et al., 2019). Differentiation protocols continue to be refined, and protocols describing the derivation or indeed forward programming of adult-like VSMCs are eagerly awaited.

### *In vitro* Models: A Simplified System

Vascular smooth muscle cells grown in 2D monoculture provide a reductive snapshot of the disease. VSMCs in the aorta are normally in contact with adventitial fibroblasts, other VSMCs in the medial lamellae and ECs lining the lumen. ECs are also closely-associated with microfibrils via integrins, and like VSMCs can also secrete fibrillin-1, although the extent and functional significance of this has not been extensively characterized (Weber et al., 2002; Rossi et al., 2010). Intimal ECs experience direct shear stress and can modulate the function of VSMCs by releasing vasoconstrictors or relaxants (Lilly, 2014). Paracrine signaling and physical interactions between ECs and VSMCs are essential for vessel development and homeostasis of mature vessels, regulating tone, blood pressure, and response to injury (Lilly, 2014). For example, endothelial signaling of TGF-β and Notch regulates VSMC phenotype and differentiation (Domenga et al., 2004; Jakobsson and van Meeteren, 2013). VSMC monoculture therefore neglects these potentially important cellular interactions, limiting the information available from such systems.

While the majority of studies investigating aortopathies focus on VSMCs, abnormalities in EC function have also been reported. NO is produced from ECs and regulates vascular tone by inhibiting VSMC contraction. MFS thoracic aortas showed differential relaxation curves in response to endothelial NO compared to wild-type controls, whereas the response in the abdominal aorta was similar for MFS or control (Chung et al., 2007). A mouse model of TAAs found that NO is implicated in TAA disease progression, where various models of TAA, including MFS, had improved aortic phenotypes when treated with NO synthase inhibitor L-NAME (Oller et al., 2017). Recently, cell-specific deletion of the *AGTR1* was investigated in a severe model of MFS (Galatioto et al., 2018). The authors found that while there was no effect with VSMC-specific deletion of *AGTR1* on disease end-points, specific ablation in ECs improved survival and decreased aortic diameter. This study highlighted that there are differential responses of ECs and VSMCs to cytokines and growth factors. This characteristic could be an important consideration for *in vitro* drug screens and discovery; once an interesting target has been identified, the response of ECs should also be studied prior to validation *in vivo*, as ECs clearly impact the disease mechanism in MFS, and likely other TAADs. This can be done in a variety of ways – ECs and VSMCs can be assessed independently or in 2D co-culture, which provides a simple way of studying both cell types together (Fillinger et al., 1997; Hastings et al., 2007). After co-culture, ECs can be purified using magnetic beads coated with anti-CD31 allowing separate downstream analysis of ECs and VSMCs (Wallace et al., 2007).

Hemodynamic forces within the blood vessel influence VSMC phenotype and function. VSMCs are not normally exposed to luminal blood flow, but instead experience low transmural interstitial flow, with cells closer to the intima experiencing greater force (Shi and Tarbell, 2011). *In vitro*, flow was found to increase VSMC contraction (Civelek et al., 2002), and induces alignment of cells perpendicular to the direction of flow (Lee et al., 2002). Studies using VSMCs alone have conflicting reports on the effect of flow on VSMC phenotype (Papadaki et al., 1996; Ueba et al., 1997; Haga et al., 2007; Shi et al., 2010), possibly due to varied forces and culture conditions. However, when VSMCs and ECs are co-cultured with shear stress, VSMC phenotype was found to be more contractile and with gene expression signatures closer to that of primary cells (Tsai et al., 2009; Collado et al., 2017).

The power of a 3D approach in HGP has been illustrated by the use of TEBVs generated from patient-derived iPSCs (Atchison et al., 2017; Abutaleb and Truskey, 2020). These TEBVs recapitulated the disease phenotypes and helped to elucidate the role of both VSMCs and ECs in disease progression. Both vasoconstriction and dilation were affected and increased medial wall thickness, calcification and apoptosis were observed. Furthermore, this 3D model was used for drug testing, where they demonstrated that the rapamycin analog everolimus increased vasoreactivity and improved VSMC differentiation. Further refinement of this model using both iPSC-derived ECs and VSMCs demonstrated that ECs are likely responsible for the abnormal response to shear stress (Atchison et al., 2020). Together, these studies highlight the importance of contributions of ECs and shear stress to VSMC biology.

When investigating aortopathies, co-culture and/or 3D approaches could be considered. While these methods provide the possibility of analyzing cells in a more native-like state, they are also more complicated, time-consuming to set up and require careful construction. A blood vessel wall contains multiple cell types, with distinct interactions being critical for their proper function. Hence, consideration of the relative ratios of VSMC, ECs and fibroblasts is required, as these can impact a number of properties including ECM deposition and modulation of VSMC phenotype (Lilly, 2014; Kuwabara and Tallquist, 2017). The arrangement and orientation of these cell types should also be considered, such that the natural hierarchy of cells forming the vasculature is respected. Inappropriate integration of these cell types could be detrimental for building an accurate disease model, obscuring critical differences between control and disease models. Finally, as we’ll discuss below, generating large amounts of iPSC-derived VSMCs can by itself be a laborious and time-consuming task; additional differentiations to ECs or set-up to create 3D systems could be difficult to accommodate in large scale.

Despite efforts to improve fidelity of iPSC-based models, the same pitfalls for any *in vitro* model remain. They lack key features provided by *in vivo models*, including involvement of the immune system and integration of complex physiological networks. We would like to emphasize that these iPSC models do not replace *in vivo* studies; instead, they complement and can accelerate the study of disease by providing a flexible platform for testing and screening. We therefore propose that with the current limitations, simple VSMC-based assays and screens in 2D could identify interesting mechanisms and targets, which can then be tested in a more complex, *in vitro* system before transitioning to *in vivo* models.

### Scale-Up and Variability Issues

Hurdles facing iPSC-based disease modeling include difficulties in scaling up production of cells and variability between differentiations. There are physical limitations to manually culturing multiple lines of iPSCs and producing large amounts of cells. Currently, aortic disease modeling is done with a handful of patient lines and controls, with assays which don’t typically require large amounts of cells (Table 1). However, for modeling diseases using 3D methods, such as TEBVs or vascular rings, many millions of cells will be required. While we discussed the ability to create virtually any mutation in the lines, the sheer number of hours and hands required to culture many different cell lines could be inhibitory, let alone deriving large quantities from each line. VSMC-derivation protocols are currently multi-step procedures, which go through an intermediate or a VSMC precursor. In addition, protocols can also include a maturation step, where cells are cultured for up to a month to accrue their phenotype. As a result, when employing such protocols, a single line will yield four distinct cell-types to monitor and manage: iPSC, intermediate/precursor, immature VSMC and mature VSMC. In our experience, given the tiered nature of the VMSC differentiation protocols, creating good intermediates is essential to producing reliable and mature VSMCs, and their maintenance should not be neglected. The length of these protocols also means that there is more opportunity for variability in differentiations. Another complication is that different iPSC lines can also behave very differently, even among control or healthy iPSC lines; skill and experience are needed to ensure that all lines are appropriately handled during differentiation in order to reduce noise from interline variability. For example, a disease model line could have abnormal proliferation and the researcher must take this into account when deciding when to passage them.

How consistently can iPSCs be differentiated by the investigator, their colleagues or even other labs using the same protocol? Considerable variation in differentiations has been reported in various fields; for example, a multi-site analysis found substantial heterogeneity in neuronal differentiations between sites using the same lines and protocol (Volpato et al., 2018). Even within research groups, variation between lines and differentiations were observed for both EB and monolayer differentiations (Osafune et al., 2008; Hu et al., 2010). When studying the 9p21 vascular risk variant, multiple iPSC lines from the same patient or even the same line differentiated multiple times exhibited considerable transcriptional variability at both iPSC and VSMC stages (Lo Sardo et al., 2018). These findings underline the concern with regard to reproducibility of data. We certainly observe differences in VSMC differentiation between individuals in our group, stressing the influence the investigator has on the final outcome. Other researchers have also observed different levels of SM-MHC^+^ cells using the same protocol or have had to modify the protocol to obtain sufficient maturity in their hands (Cheung et al., 2012; He et al., 2018; Trillhaase et al., 2018; Zhang et al., 2019). These differences could be due to the use of different iPSC lines, but are likely also impacted by variation imparted by the user. Current iPSC models of aortic disease are focused on severe models of disease. However, when modeling the effects of a milder mutation or variant, the effect of genotype may not be observed if the differentiations themselves are highly variable.

A common issue we’d like to highlight for many differentiation protocols is the use of non-chemically-defined media and coatings, such as serum or Matrigel, and the reliance on cytokines where different batches of these reagents may have varying effects on differentiated cells. Currently in disease modeling, serum is used to stimulate growth of VSMCs in various protocols after differentiation (Table 2), and high levels of serum are known to result in loss of contractile phenotype (Alexander and Owens, 2012). Aside from the use of Matrigel, a near chemically-defined protocol to generate VSMCs has been developed (Patsch et al., 2015) and modified protocols have recently been used to model HGP (Atchison et al., 2020) and LDS (Gong et al., 2020). In addition, many VSMC protocols rely on growth factors, such as TGF-β and PDGF-BB for differentiation. While these protocols do work, investigators should be wary of the numerous factors which may influence the efficacy of these cytokines, such as storage method and batch-to-batch variation. In the cardiac field, a protocol using entirely chemically-defined media to produce cardiomyocytes was developed by systematically assessing the necessity of individual factors (Burridge et al., 2014). Interestingly, they found that only three components were crucial for cardiomyocyte differentiation. This protocol resulted in improved consistency of differentiations in the 11 iPSC lines that were tested. In addition to ease and consistency, this approach could also enable researchers to scale-up production more than is possible using cytokine and xeno-containing formulations. Similar advances have been made in other fields (Erceg et al., 2008; Touboul et al., 2010) and would be beneficial in advancing aortic disease modeling.

It goes without saying that new protocols have to be carefully assessed and compared with tissue or primary cells to ensure that that the stem cell-derived product has the correct identity. With advances in the past decade, decreasing price and availability of large-scale experiments (Hasin et al., 2017), detailed comparisons can be performed to assess the quality and consistency of differentiation protocols. This was an approach demonstrated by Patsch et al. (2015), where they showed high correlation between their differentiated and primary VSMCs using both transcriptomics and metabolomics. In addition, high-throughput “omics” can be used to assess the consistency of differentiations (Paull et al., 2015), and single-cell RNA sequencing has been used to identify pivotal steps in differentiation protocols (Chu et al., 2016; Han et al., 2018). We predict that future iterations of protocols will utilize these tools to help direct and objectively assess the quality of differentiation protocols.

**FIGURE 3:**
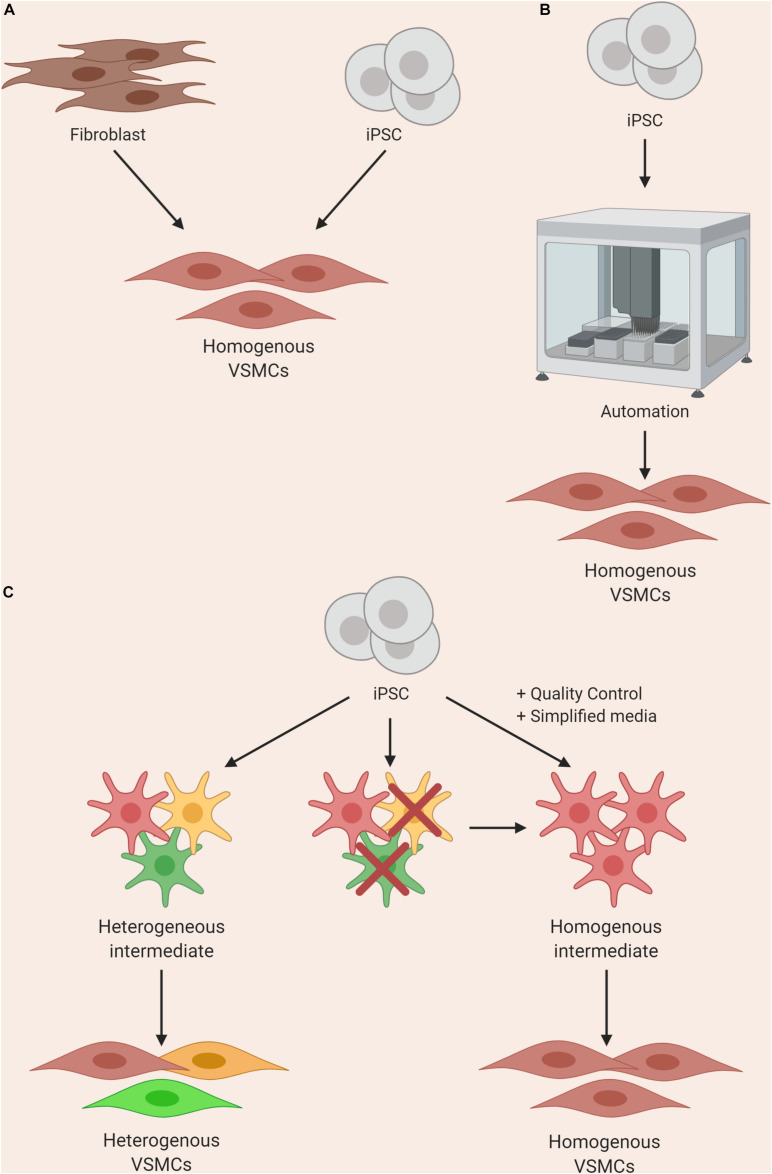
Approaches to improving homogeneity of VSMC differentiations by using **(A)** direct or forward reprogramming methods, **(B)** automation, or **(C)** improved quality control and simplification of media components.

Alternative approaches, such as direct reprogramming and forward reprogramming, may circumvent the imperfect approximations of developmental pathways used for typical differentiation protocols, and reduce the number of intermediates required (Figure 3A). Work on direct reprogramming has been shown in various fields (Kelaini et al., 2014), including the derivation of cardiomyocytes from fibroblasts (Ieda et al., 2010). Forward reprogramming has been demonstrated to rapidly convert hESCs into neurons, skeletal myocytes, and oligodendrocytes by overexpressing key lineage-specific transcription factors (Pawlowski et al., 2017). These approaches in VSMCs have only recently been reported, and warrant further investigation (Yeung et al., 2017; Hirai et al., 2018). In addition, it may be challenging to produce the significant region-specific VSMCs using these strategies with our current limited understanding of the fundamental differences between VSMC from varying embryonic origins.

Until differentiation methods are refined, steps can be taken to improve reliability of current protocols with clearly-defined parameters for quality control at various stages. For example, stringent quality control should be performed after the derivation of an intermediate state before inducing cells toward a VSMC fate (Cheung et al., 2014); if the cells fail to meet the set criteria, they should not be used for further differentiation (Figure 3C). In addition, when VSMCs are produced, analysis of markers and/or function should be routinely assessed. These criteria should ideally be shared with collaborators in order to reduce the site-specific variability as described by Volpato et al. (2018). Furthermore, identification of novel surface markers exclusively expressed on contractile and mature VSMCs could be used for cell sorting and/or quality control. Lastly, wherever possible, the use of multiple iPSC clones from the same patient could also improve the signal-noise ratio, as different clones can themselves be highly variable (Lo Sardo et al., 2018; Popp et al., 2018).

Automated systems and machine learning could significantly reduce the input needed from the researcher when culturing multiple lines, improving consistency and enabling increased production. Automated iPSC culture systems have been developed and would present a solution to the workload and variability problems (Conway et al., 2015; Paull et al., 2015; Figure 3B). The method developed by Paull and colleagues describes the capacity to reprogram, expand and characterize hundreds of lines per month with significant reductions in reagent cost. In addition, transcriptomics analysis indicated that there was a significant reduction in variability in EB assays when compared to manual processing. This system was put to the test when iPSCs were differentiated into dopaminergic neurons using a 30-day protocol and the resulting cells maintained expected marker expression. This automated system was utilized by another group for cardiomyocyte differentiation, and found success with producing a maximum of 3 × 10^9^ cardiomyocytes per batch (Denning et al., 2016). A recent method describing high-yield derivation of VSMCs based on an existing protocol (Patsch et al., 2015) was described, where VSMCs were derived in alginate hydrogel tubes (Lin et al., 2019). This method yielded 5 × 10^8^ cells/ml in 10 days; as a result, bioengineering methods could rely on such advances for producing high numbers of cells.

The behavior of some patient lines with certain mutations can be tremendously divergent compared to control lines, requiring careful assessment from an experienced researcher to consider not only cell density, but also morphology, heterogeneity and survival. In our experience working with MFS patient iPSC lines, when deriving NC-VSMCs, the cells steadily exhibit more of the disease phenotype throughout the course of differentiation. They require much closer monitoring and the resulting differentiations can be more heterogeneous compared to controls, due to varied cell density caused by increased apoptosis and slower proliferation. Innovations in robotics and machine learning could overcome these bottlenecks. For example, machine learning has been developed to identify cells in phase contrast based on morphology alone without the need for molecular labeling (Kusumoto and Yuasa, 2019). This technology, in conjunction with modular automated systems, could be powerful for processing large numbers of iPSC lines, including cells derived from severely affected lines, as it could potentially remove the need for an experienced “eye” when culturing cells. However, at the moment, the protocols and technologies are not yet compatible with one another for robust, automated systems; the labor-intensive manual culture and differentiation of iPSC lines into VSMCs are current limitations for large-scale studies.

### Conclusion

Induced pluripotent stem cell-based modeling of aortic disease is still relatively new, with only a handful of papers describing disease models (Table 1). Despite the practical advantages of using this system, there are limitations. Most notably, the cells obtained from differentiation are not as mature as VSMCs in tissue due in part to absent mechanical cues, lack of contact with ECs and other physiological signals. In addition, without appropriate quality control, variability between differentiations can result in noisy and inconsistent data. Large-scale experiments involving multiple lines are difficult to perform as manual passaging and differentiation is required as a result of the complexity of certain protocols. In spite of this, we are certain that continued refinement of differentiation protocols and technological advances will be able to overcome these limitations to create valuable tools for understanding, preventing and treating aortopathies.

## Potential and Future Directions

### Regenerative Medicine

The first engineered blood vessel was a relatively simple construct made from collagen and primary bovine VSMCs, which was then lined or coated with primary ECs and adventitial fibroblasts, respectively (Weinberg and Bell, 1986). Since then, efforts have been made to produce clinically-relevant TEBVs with the required mechanical specifications, as reviewed by (Kumar et al., 2011). Recently, tissue engineered vascular grafts (TEVGs; Carrabba and Madeddu, 2018; Song et al., 2018) and vasculature-on-a-chip (Kim et al., 2017) models have been developed to accommodate the gold standard properties of a transplantable graft using either self-assembling bioprinting technology or using natural or synthetic scaffolding (Konig et al., 2009; Wise et al., 2011). These models have the properties of a successful graft, such as an autologous endothelium, anti-thrombogenic properties and minimum integrity span of 21 months, with appropriate permeability, compliance, elastic modulus, and a minimum burst pressure of 1700 mmHg (Konig et al., 2009).

The use of TEVGs in regenerative medicine is still under development, with many groups innovating with novel ways to tackle the problems facing engineered grafts. For example, grafts comprising decellularized ECM on biodegradable scaffolds have been suggested to serve as readily available TEVGs; these have been tested in a variety of animals models (Dahl et al., 2011) and can exploit recent advances in 3D tissue printing to provide patient-specific grafts (Fukunishi et al., 2017; Best et al., 2018). Cell-free vessel grafts have been generated by allowing cells to secrete ECM for longer periods to more closely mimic the *in vivo* environment and are then decellularized (Lawson et al., 2016; Row et al., 2017). Furthermore, functionalization of TEVGs with biological signals such as the angiogenic cytokine VEGF have been shown to trigger *in situ* tissue endothelial regeneration (Koobatian et al., 2016). Although advances in traditional translational approaches for cardiac anomalies have paved the way for regenerative medicine, these TEVGs still suffer from a number of common issues including insufficient patency, integration, hemodynamics, immune-compatibility with the graft cell source and mechanical strength, as outlined by others (Pashneh-Tala et al., 2016; Matsuzaki et al., 2019; Skovrind et al., 2019).

Currently, if a TAAD patient’s aorta dilatates sufficiently, prophylactic surgical intervention is required. iPSC-based systems raise the possibility of developing regenerative cell therapies for patients with aortic disease, where TEVGs can be produced from patient iPSCs. In addition, the availability of gene editing tools means that the TAAD-causing mutation(s) can be corrected in a patient’s iPSCs. These, in turn, could be differentiated into VSMCs and developed into a healthy TEBV, to be used as an autologous bio-compatible graft. Furthermore, patient-derived iPSCs would provide immune-compatible grafts. These would be particularly useful in pediatric patients where cardiovascular grafts would ideally grow in line with the patient’s normal growth and development (Sugiura et al., 2018). To our knowledge, there have been limited applications of iPSC-based TEVGs, let alone in the context of aortic disease. In one case at least, TEVGs demonstrated mechanical strength comparable to that of native veins; when implanted in rats, they showed sustained mechanical function and patency (Sundaram et al., 2014; Luo et al., 2020). While the application of iPSC-derived VSMCs in regenerative medicine for the treatment of aortic disease is attractive, we would like to caution that this represents a very labor-intensive task. We discussed earlier the current difficulties in obtaining large numbers of consistently-differentiated VSMCs. In addition, the approaches highlighted above would need to be tailored to each individual patient. In our experience, establishing and characterizing a new iPSC line can take weeks before differentiations can be started, which can themselves take up to a month before TEBV construction can begin. The timeline grows even longer if gene editing also has to be involved. As an alternative, haplotype matched/allogenic iPSCs, MSCs or ESCs could be used providing the advantage of well-defined VSMC differentiation protocols but without needing to develop individual lines and grafts specifically for each patient (Sundaram et al., 2014; Gui et al., 2016; Elliott et al., 2019; Luo et al., 2020). These can be prepared in a variety of formats, including printed, electrospun, or decellularized scaffold grafts. This approach could be developed even further by the use of lineage-specific protocols to create the closest approximation possible of on-demand TEVGs, catering to different matrix compositions.

### Prediction of Disease Severity and Phenotype-Genotype Correlation

Aortopathies have profound effects on the life quality of affected patients; not being able to know what the severity of the disease is can be an enormous burden. This is complicated by the lack of understanding of genotype-phenotype correlation in many TAADs – even within families, disease severity can vary significantly. This is even more difficult in sporadic cases, where there is no family history to infer prognosis from. The best solution at the moment is to monitor the patient’s aorta by cross-sectional imaging, administer anti-hypertensives and intervene with surgery if the dilatation exceeds a threshold. However, what if we were able to predict the patient’s disease severity and likely progression?

In MFS, there is high inter- and intra-familial variation in patients. *FBN1* is a large gene, encoded by 65 exons, with over 3,000 mutations identified to date (Collod-Béroud et al., 2003). Aside from neonatal MFS, there may be some broad genotype-phenotype correlation with *FBN1* mutations; in MFS, mutations in exons 24–32 or premature terminations are associated with a more severe disease outcome with cardiovascular complications (Faivre et al., 2007). Disease-causing mutations of *FBN1* can be categorized as dominant-negative or haploinsufficient. In dominant-negative forms, the mutant product interferes with normal microfibril formation or is mis-incorporated. Various studies in patient fibroblasts have found abnormalities with reduced synthesis, delayed intracellular processing, and secretion (Aoyama et al., 1994; Schrijver et al., 1999; Whiteman and Handford, 2003). Haploinsufficiency is typically caused by mis-sense or frameshift mutations; analysis of patient fibroblasts found a reduction in the mRNA levels of mutant fibrillin-1, and a disproportionately low amount of fibrillin-1 deposition (Schrijver et al., 2002). Large studies have concluded that mutations causing haploinsufficiency of fibrillin-1 resulted in a 2.5-fold increase in the risk of cardiovascular death compared to dominant-negative mutations (Franken et al., 2016), and that mutations involving cysteines tend to also result in more severe clinical presentations (Aubart et al., 2018).

Although these broad associations may explain in part some of the variation in disease severity observed between patients with different mutations, it is unclear what factors contribute to variation *within* families or between patients with the same mutation in different families. Variation in genetic background clearly plays a key role in the different expression of disease. However, identifying clear associations between genotype and phenotype can be challenging for rare diseases due to the statistical power needed to identify gene modifiers in population genomics. MFS is the most common TAAD, with an incidence for 1 in 5000, whereas diseases such as LDS and vEDS are even rarer. A small study in patients with TAAs identified that variants in *ADCK4* and *COL15A1* were associated with mild disease (Landis et al., 2017). Recent studies have shown that integrating multiple methods can overcome limitations of studying rare disorders (Aubart et al., 2018). Whole-exome sequencing and association studies in a large cohort of 1070 patient fibroblasts has identified interesting mutations and variants accompanying a more severe presentation of MFS (Aubart et al., 2018). Severe cases of MFS were associated with co-occurrence of another TAAD-causing mutation, including additional variants of *FBN1* or *SMAD3*. Interestingly, severe disease was also associated with mutations in *COL4A1*; variants of *COL4A1* have been reported in stroke and cerebral aneurysms (Lanfranconi and Markus, 2010). Three major modifier regions were identified, corresponding to loci encoding *ECE1*, *PRKG1* and *MMPs*.

iPSC-based modeling could help with severity prediction in two ways – first, by deepening our understanding of the genetic variants interacting with disease-causing mutations, and second, by potentially providing a platform with which to assess patient-specific disease severity. Whole-exome sequencing of a patient’s genome could give clinicians an initial idea of the expected disease severity, based on the risk variants present. These identified variants could then be introduced into various iPSC lines to further underpin their role in modulating disease. This can be done in a variety of patient lines, isogenic controls and also in healthy iPSC lines. This approach was used in an investigation of metabolic disorders, where variants previously discovered using genome-wide association studies were investigated using patient iPSCs (Warren et al., 2017). From patient iPSCs, simple cell-based assays can be employed to construct a prediction of clinical severity in the patient. In the case of TAADs, this could be looking at proteolytic activity, abnormal ECM deposition or cell death. Guidelines for determining *in vitro* disease severity can be developed through iterative empirical testing until these *in vitro* benchmarks are sufficiently refined and can be robustly linked to clinical severity. This predictive tool could then be used in conjunction with clinical benchmarks to provide a more informed prognosis. Together, these methods could be used to predict the course of the disease and guide treatment for patients.

### Drug Screens and Precision Medicine

Patient-derived VSMCs can be subjected to drug testing to identify compounds which ameliorate function. The ease of assays in 2D culture systems makes it feasible to use multi-well formats, test their response to various drugs and analyze a range of readouts, including VSMC contraction, proliferation, and secretome. For example, multiple iPSC lines from a hypertensive pharmacogenomics cohort were differentiated to functional VSMCs and their responses to contractile agonists and inflammatory cytokine TNF-α were analyzed (Biel et al., 2015). This work established robust high throughput assays for pharmacogenomics studies, paving the way for future studies which may incorporate the use of isogenic controls. A recent report of a model for SVAS has used an iPSC model to test the effect of different classes and combinations of drugs, finding that mTOR inhibitor everolimus was the most effective at rescuing the disease phenotype (Kinnear et al., 2020). Interestingly, they found that combination therapy using everolimus and additional classes of drugs was not beneficial. As emphasized earlier, interesting drug targets identified from large-scale screening can then be tested in a more complex and physiological set-up, possibly incorporating shear stress and co-culture systems to better mimic the aorta (Collado et al., 2017), preferably using lineage-specific cells where possible. Indeed, Atchison and colleagues have developed a 3D model of HGP from iPSC-derived VSMCs to test drug toxicology efficacy and dose response for various drugs (Atchison et al., 2017).

Thoracic aortic aneurysm and dissections are chronic and life-long conditions. Although establishing, characterizing, creating isogenic controls and finally differentiating new patient lines is a laborious task, drug testing and personalized medicine for diseases such as TAADs would be a worthwhile investment for the patient. With advances in automation, machine learning and refinement of existing protocols, we predict that this entire process of patient-specific drug screens and personalized medicine will be streamlined and simplified. Furthermore, developments in vascular 3D modeling to reduce costs, variability and intricacy may eventually allow for high-throughput drug screening in 3D. In addition to therapies and precision medicine, another way in which iPSC modeling could be beneficial would be to test for vascular toxicology. These sorts of studies have been performed in the cardiac field (Zhang et al., 2012; Florido et al., 2017; Sharma et al., 2017). Given that the cardiovascular complications of diseases such as MFS can be fatal, it may be worthwhile to undertake toxicology studies on additional drugs that could be detrimental to aortic health. For example, based on research focused on tendon rupture, the commonly used quinolone antibiotics are thought to cause connective tissue defects by upregulating MMP expression (Sendzik et al., 2010; Tsai et al., 2010). Their use in an animal model of TAAD (LeMaire et al., 2018) and susceptible patients (Daneman et al., 2015; Lee et al., 2015; Pasternak et al., 2018; Noman et al., 2019) is associated with a higher risk of complications and they are no longer recommended for patients with aortic disease. Both established and new drugs could be screened in iPSC models to identify those that pose risks to patients with aortopathies.

### “Clinical-Trials-in-a-Dish”

**FIGURE 4:**
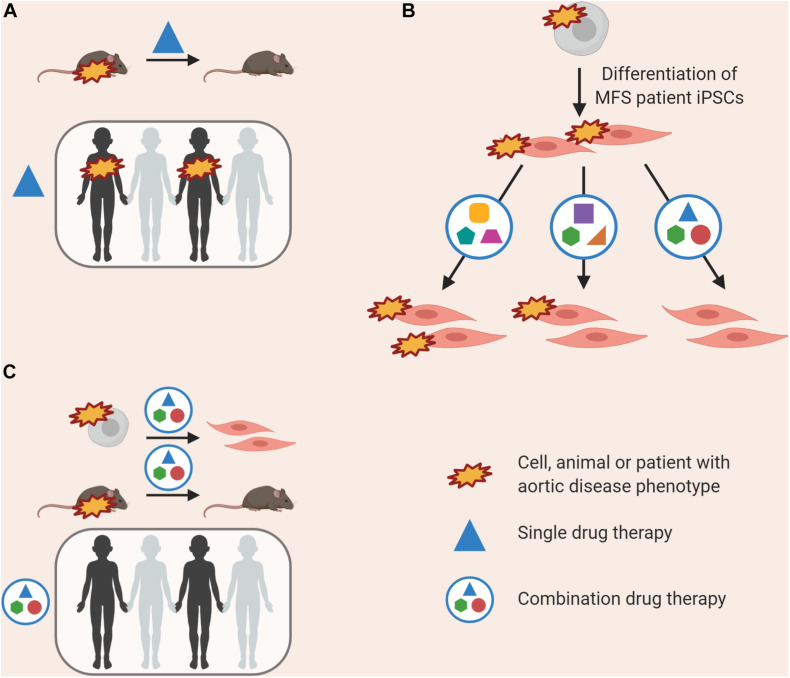
Currently, successful use of a drug in animal models is the prerequisite for use in clinical trials **(A)**; this may lead to an amelioration in disease phenotype in some individuals, but not all. “Clinical-trials-in-a-dish” can be performed, where the effects of a combination of drugs at low doses is tested on patient-derived VSMCs, allowing us to target multiple de-regulated pathways **(B)**. This combination therapy could then be validated in rodent models prior to use in clinical trials, and may have an effect in more patients **(C)**.

Induced pluripotent stem cell models can provide guidance for future clinical trials (Figure 4). In the case of the various losartan clinical trials, while some patients may have responded well to treatment with losartan, noise from non-responders would render such data non-significant despite the success in mice (Figure 4A). This may be due to the nature of mutation in *FBN1*, disease severity, genetic background, age of treatment or contribution from all of the above. Prior to a clinical trial, pre-screening patient-derived VSMCs to identify the pathways that are likely deregulated in the cohort, or conducting a preliminary trial *in vitro* before the full trial involving patients could be valuable (Figure 4B).

A multiplicity of signaling abnormalities has been found in MFS. We and others have identified that other non-canonical TGF-β signaling pathways are altered in MFS, including ERK and p38 (Carta et al., 2009; Habashi et al., 2011; Granata et al., 2017; Sato et al., 2018), and it is well-established that patient disease severity ranges widely. Other groups have identified a role for NO signaling contributing to the disease (Chung et al., 2007; Oller et al., 2017). How do we reconcile the multiple signaling abnormalities seen in this condition with disease pathophysiology? We propose that multiple pathways may be deregulated downstream of a single *FBN1* mutation and that these may also be deregulated to different extents. Using iPSC-derived VSMCs, “clinical-trials-in-a-dish” involving multiple drugs at tolerable, clinically-relevant concentrations can be employed before introducing the best combination in clinical trials (Figure 4C).

## Conclusion

There is no doubt that iPSCs and the ability to generate human disease models offer a powerful new weapon in our armamentarium against thoracic aortic diseases. In this review we have presented the current state-of-the-art and highlighted how this technology is being used to tackle critical questions in the field. A key strength of iPSC-based disease modeling is its link to individual patients, which encapsulates genetic variants or mutations in the context of a disease-susceptible genetic background. Rapid developments in differentiation protocols, including the ability to generate lineage specific VSMCs, have facilitated robust *in vitro* models. Together with ease of genetic modification, these models allow us to increasingly clearly delineate pathological mechanisms and carry out drug screening to develop much-needed new therapies for aortic disease.

We have tried in this review to offer our personal insights into the details and nuances of establishing iPSC-based *in vitro* disease models of aortopathies. We have also highlighted the challenges and limitations of such an approach, such as limited cell types and lack of 3D structure and blood flow, where appropriate. Despite the challenges, we are excited by the scientific and therapeutic opportunities presented by these model systems and particularly for future developments such as deeper genotype–phenotype analyses, vascular toxicology studies, “clinical trials-in-a-dish,” and precision medicine – potentially enabling better tailoring of therapy to individuals.

## Author Contributions

HD, DS, and SS: writing, reviewing, and editing of manuscript. All authors contributed to the article and approved the submitted version.

## Conflict of Interest

The authors declare that the research was conducted in the absence of any commercial or financial relationships that could be construed as a potential conflict of interest.

## Abbreviations

α-SMA: α-smooth muscle actin
AT1R: Angiotensin II receptor, type 1
BAV: Bicuspid aortic valves
EB: Embryoid body
EC: Endothelial cell
ECM: Extracellular matrix
ESC: Embryonic stem cell
FC: Flow cytometry
HGP: Hutchison-Gilford progeria
IF: Immunofluorescence
iPSC: Induced pluripotent stem cell
KSR: Knock-out serum replacement
LDS: Loeys-Dietz syndrome
LM: Lateral plate mesoderm
MFS: Marfan syndrome
MMP: Matrix metalloproteinase
NC: Neural crest
NO: Nitric oxide
NR: Not reported
PM: Paraxial mesoderm
SMGM: Smooth Muscle Growth Medium
SM-MHC: Smooth muscle myosin heavy chain
SPC: Sphingosylphosphorylcholine
SVAS: Supravalvular aortic stenosis
TAAD: Thoracic aortic aneurysm and dissection
TEBV: Tissue-engineered blood vessel
TEVG: Tissue-engineered vascular graft
TGF: Transforming growth factor
T β RII: TGF- β receptor II
vEDS: Vascular Ehlers-Danlos syndrome
VSMC: Vascular smooth muscle cell.
